# Direct and Indirect Antimicrobial Activities of Neuropeptides and their Therapeutic Potential

**DOI:** 10.2174/138920312804871139

**Published:** 2012-12

**Authors:** Daria Augustyniak, Judyta Nowak, Fionnuala T Lundy

**Affiliations:** 1 Department of Pathogen Biology and Immunology, Institute of Genetics and Microbiology, University of Wroclaw, Przybyszewskiego 63/77, 51-148 Wroclaw, Poland; 2 Centre for Infection and Immunity, School of Medicine, Dentistry and Biomedical Sciences, Queen’s University Belfast, 97 Lisburn Road, Belfast, BT9 7AE, Northern Ireland, UK

**Keywords:** Neuropeptides, anti-infective action, antimicrobial action, immunomodulation, therapeutic potential.

## Abstract

As global resistance to conventional antibiotics rises we need to develop new strategies to develop future novel therapeutics. In our quest to design novel anti-infectives and antimicrobials it is of interest to investigate host-pathogen interactions and learn from the complexity of host defense strategies that have evolved over millennia. A myriad of host defense molecules are now known to play a role in protection against human infection. However, the interaction between host and pathogen is recognized to be a multifaceted one, involving countless host proteins, including several families of peptides. The regulation of infection and inflammation by multiple peptide families may represent an evolutionary failsafe in terms of functional degeneracy and emphasizes the significance of host defense in survival. One such family is the neuropeptides (NPs), which are conventionally defined as peptide neurotransmitters but have recently been shown to be pleiotropic molecules that are integral components of the nervous and immune systems. In this review we address the antimicrobial and anti-infective effects of NPs both *in vitro* and *in vivo* and discuss their potential therapeutic usefulness in overcoming infectious diseases. With improved understanding of the efficacy of NPs, these molecules could become an important part of our arsenal of weapons in the treatment of infection and inflammation. It is envisaged that targeted therapy approaches that selectively exploit the anti-infective, antimicrobial and immunomodulatory properties of NPs could become useful adjuncts to our current therapeutic modalities.

## 1 INTRODUCTION

**Fig. (1):**
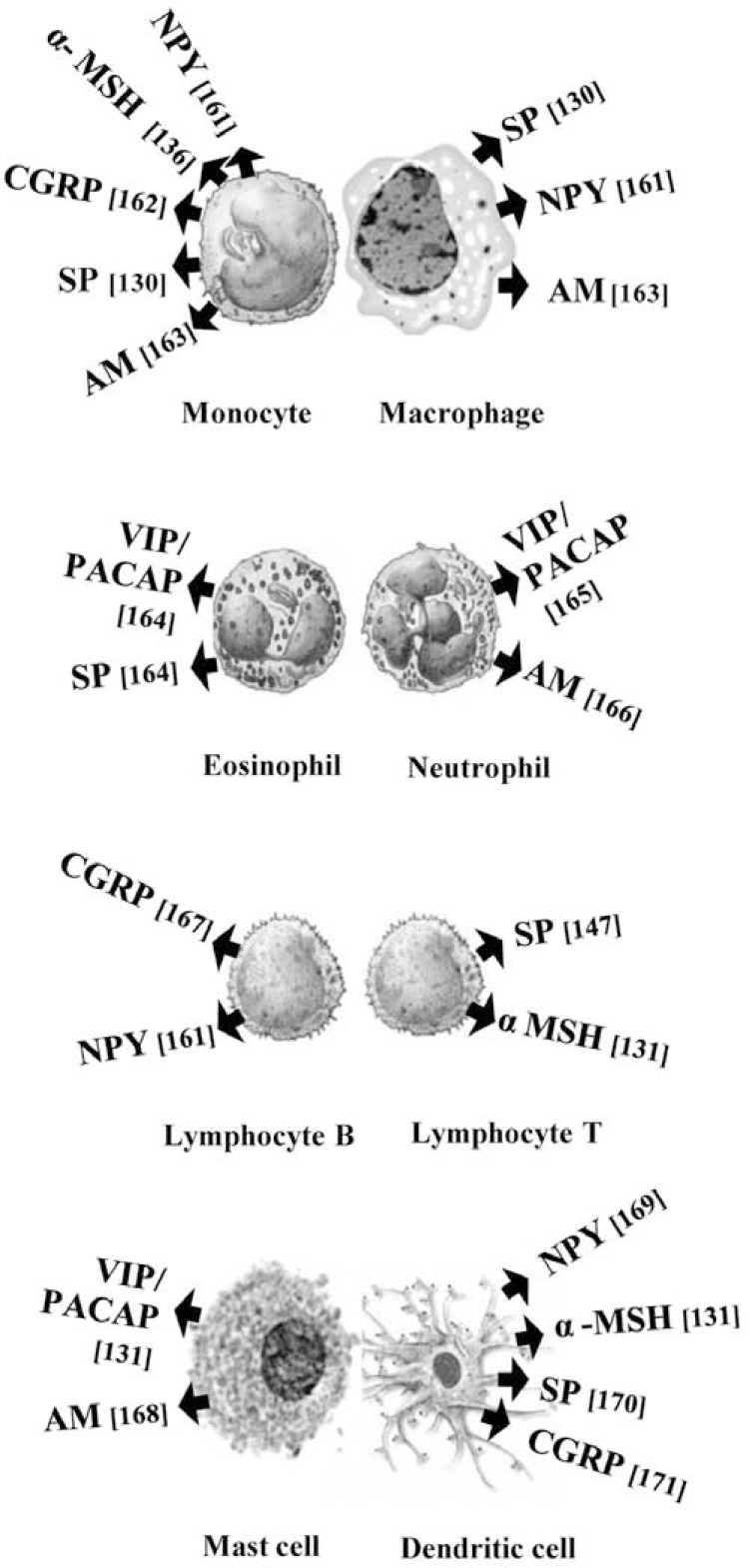
Production of neuropeptides by cells of the human immune system.

Human host defense peptides, also known as antimicrobial peptides, belong to a class of molecules expressed predominantly at host-environment interfaces such as the oral cavity, respiratory tract and gastrointestinal tract. In principle, the distribution of these evolutionarily ancient molecules within ‘portals of entry’ for microbial invasion supports their antimicrobial function [1]. In the past decade some neuropeptides (NPs) have been associated with this group of peptides and it has been postulated that NPs contribute to the formation of local barriers of defense against pathogens. The traditional roles described for NPs are signal transmission and modulation in the central and peripheral neural systems. However the release of NPs from the peripheral nerve fibers of innervated organs, resident tissue cells or infiltrating immune cells suggests they may also have potential roles in the local microenvironment, (Fig. **(1)**). Prospective roles for NPs in the local tissue microenvironment include those generally associated with conventional host defense peptides such as direct antimicrobial action against microorganisms or immunomodulatory effects on host cells. Drawing analogies from research on conventional host defense peptides, a direct antimicrobial effect for a given NP could potentially be observed if: (i) the NP is released at sites of microbial invasion; (ii) the NP gains direct access to the target micro-organism; (iii) the microbial membrane can be perturbed leading to microbial death in the presence of the NP and (iv) the concentration of NP present in the local microenvironment produces an antimicrobial effect *in vitro* [2,3]. An immunomodulatory action for a given NP could be established if: (i) there is an association between specific nerve fibers and primary or secondary lymphoid tissue; (ii) the released NPs are available to immune cells expressing the appropriate G-protein coupled NP receptors and (iii) the immunoregulatory effect of the NP is confirmed *in vitro* or *in vivo* [4]. 

NPs and host defense peptides share several structural and biophysical characteristics, despite their physiological and source diversity. These features include low molecular mass (<10 kDa), cationicity and amphipathic design. These properties enable NPs to interact with the negatively charged components of the microbial cell envelope, leading to disturbances in membrane barrier function, and ultimately microbial cell lysis and death [5]. In our quest to develop novel antimicrobials, it is important to study the entire spectrum of naturally occurring human peptides with potential roles in host defense and exploit these molecules as therapeutics for combating infection. In this respect, detailed knowledge of the antimicrobial actions exerted by NPs, their immunomodulatory effects, the underlying signal transduction pathways they trigger, and their potential cooperation with other immune components remain to be fully elucidated. While countless NPs may be postulated to contribute to the various aspects of antimicrobial defense, this review will focus chiefly on neuropeptide Y (NPY), substance P (SP), calcitonin-gene related peptide (CGRP), adrenomedullin (AM), vasoactive intestinal peptide (VIP) and melanocyte-stimulating hormone (α-MSH). We will summarize current data on both the direct antimicrobial and indirect immunomodulatory effects of these NPs and critically discuss their potential therapeutic use.

## 2 THE DIRECT ANTIMICROBIAL ACTIVITY OF NEUROPEPTIDES

### 2.1 Mechanism of Action

Numerous studies have reported direct antimicrobial [6-12] and antiparasitic [13, 14] effects of NPs* in vitro*. Several mechanisms of antimicrobial peptide action have been proposed to date. However the majority of these peptides utilize membrane-disruption as an important mode of action. Like conventional antimicrobial cationic peptides, NPs may act preferentially on microbes through a sequence of events involving: (i) electrostatic binding of the positively charged NP to the negatively charged microbial surface; (ii) destabilization and disorganization of the negatively-charged phospholipid bilayer leading to disruption of microbial membrane integrity; (iii) subsequent permeabilization of the membrane and (iv) cell death by osmolysis [15]. The topological (rather than linear) amphipathic design of a peptide appears to influence its membrane permeabilization ability. As a result, charges are distributed in organized clusters on the surface of the peptide and this is fundamental for significant antimicrobial action [16, 17]. A loss of microbial membrane integrity has been confirmed for the NPs NPY, α-MSH and AM. For these NPs the antimicrobial activity, residing in the C-terminal peptide fragment, was shown to be associated with a rich combination of positively charged amino acids and hydrophobic residues [6, 18, 19]. 

Despite the confirmed membrane-disrupting mechanism of action for some NPs and other host defense peptides, a universal or simple correlation between antimicrobial activity of peptides and their structural or physicochemical features (charge, hydropathicity, transmembrane tendency etc) is not demonstrated easily [20, 21]. This suggests that peptide-membrane interactions are determined by a sensitive balance of electrostatic and hydrophobic interactions in which the superimposition of many physicochemical properties exists [22]. It has been suggested that a membrane-disrupting mechanism of action in which peptides target multiple hydrophobic and polyanionic sites in the microbial membrane, cannot easily be subverted by bacterial pathogens. The acquisition of microbial resistance appears to require substantial modification of anionic surface components to increase the number of positive charges. Complete redesign of the microbial membrane in this way is metabolically expensive and appears to be unprofitable for most pathogens. As a result, pathogen resistance to membranolytic peptides is limited compared with resistance to conventional antibiotics [23].

In addition to physical perturbation of the membrane, some peptides may exert their antimicrobial action as a result of interference with intracellular metabolic functions or with membrane-associated biosynthesis [5]. A good example is the candidacidal effect of α-MSH that binds to a fungal membrane receptor and mediate the induction of cyclic adenosine monophosphate (cAMP) [7]. It is postulated that the rise in cAMP induced by α-MSH interferes with the yeast’s regulatory mechanisms in essential signaling, leading to cell death [24]. α-MSH has been shown not only to significantly reduce the viability of *Candida albicans* but also to inhibit germ tube formation, which in turn limits its harmful transition to the virulent yeast-filamentous form. A similar inhibition of candidal hyphal development has been reported for galanin message-associated peptide (GMAP) [25]. Since it is known that conversion between yeast and filamentous forms is correlated with the virulence of *Candida albicans* [26], it is reasonable to suggest that α-MSH and GMAP have a role in blocking the adhesion and invasion of this pathogen into host cells. Interestingly, the unique effect of α-MSH on yeast adenyl cyclase activation and increased intracellular cAMP mimics its receptor-mediated effect on melanocortin receptors in mammalian cells [27]. It remains to be determined however whether the proposed fungal membrane receptors are homologs of the mammal melanocortin receptors. 

Interactions with metabolic targets are also typical for other antimicrobial peptides. For example, human histatin-5 appears to penetrate the plasma membrane of *Leishmania* in a non-lethal manner. Once inside the cell it targets the mitochondrial membrane disturbing ATP synthesis and leading to parasite death [28]. The NP AM has at least two distinct antibacterial mechanisms of action: (1) classical cell-wall disruption in *E. coli* and (2) interference with bacterial cell division and abnormal septum formation in *S. aureus* [19]. A more unusual direct mechanism of action for VIP was revealed against the protozoan parasite *Trypanosoma brucei*, the etiological factor in African sleeping sickness. Rather than inducing membrane perturbation, like a conventional antimicrobial peptide, VIP is endocytosed and accumulated intracellularly by the parasite. Thus, the trypanolytic action of VIP involves initial peptide endocytosis followed by disruption of lysosome integrity, cytosolic accumulation of glycolytic enzymes and finally NP-mediated autophagic-like cell death connected with energy metabolism failure [14, 29]. 

The examples cited above indicate clearly that NPs may use a variety of direct antimicrobial mechanisms of action. Some of them can use more than one mechanism for their microbicidal activity. The potential utilization of more than one mechanism of action could be suggested to increase the efficacy of NPs compared with conventional drugs, which generally target a single biosynthetic pathway. The direct effects of NPs could interfere with microbial pathogenesis at a number of levels. NPs could block microbial adhesion to host epithelial or endothelial cells, biofilm formation and cell invasion. However all these promising antimicrobial activities of NPs have to be confirmed *in vivo.*

### 2.2 *In Vitro* Antimicrobial Assays

A number of papers have demonstrated the direct antimicrobial activities of NPs against microorganisms and protozoan parasites (Table **1)**. The factors that influence the efficacy of a given NP against a microbial target *in vitro* depend on the physicochemical features of the peptide, its concentration and the intrinsic sensitivity of the target. The sensitivity of the target organism to antimicrobial action may be related to inherent features of the microbial membrane including the presence/absence of lipopolysaccharide (LPS), lipoteichoic acid and glycans. However, subtle differences in protocols between different laboratories, including handling of the peptide, broth composition, presence of serum in the media, inoculum size, growth phase of the micro-organism and incubation time for the experiment, will also affect results and should be taken into consideration when comparing data between research groups. 

#### 2.2.1 Species and Strains Sensitivities 

NPs display antimicrobial activities at micromolar concentrations *in vitro* against various Gram-negative and Gram-positive bacteria, yeast, fungi [6-12, 21] and protozoan parasites [13, 14]. NPs appear to be more potent against Gram-negative than against Gram-positive bacteria which is a logical consequence of the structural differences in their envelopes. In this case, the thinner peptidoglycan layer and less compact cell wall of Gram-negative bacteria may permit easier peptide access to the plasma membrane. However in some Gram-negative pathogens that produce proteases as virulence factors, resistance to NPs could arise as a result of inactivation by these enzymes. Resistance to the antimicrobial action of the neuropeptides CGRP and AM has been reported for highly proteolytic oral anaerobic bacteria such as *Porphyromonas* *gingivalis* and *Prevotella spp *[30, 31].

One of the most effective NPs against the representative Gram-positive pathogen, *Staphylococcus aureus,* appears to be α-MSH. It has been shown that α-MSH is staphylocidal against both methicillin-sensitive (MSSA) and methicillin-resistant (MRSA) strains. This finding is particularly important given that MRSA is a leading cause of nosocomial infections and is more resistant to conventional antibiotic treatment. Furthermore, staphylocidal α-MSH activity was not affected by the presence of physiological concentrations of NaCl (150 mM) or divalent cations such as Ca^2+^ (1 mM) and Mg^2+^ (1 mM) [32]. This is significant if we consider that physiological concentrations of these cations, present in the majority of body fluids, can decrease the efficacy of antimicrobial peptides. The confirmed potency of α-MSH against MRSA in physiologically-relevant salt concentrations may have beneficial therapeutic implications for the peptide against this aggressive pathogen. In addition, α-MSH has been shown to be effective against staphylococcal biofilms [18]. The biofilm mode of growth is detrimental in the pathogenesis of several bacterial driven diseases including osteomyelitis, periodontitis and chronic wound infections [33]. Since organisms in biofilm form are much more resistant to therapeutics than their planktonic counterparts, the actions of α-MSH against biofilms are worthy of further investigation as antimicrobial agents. 

Considering the results presented in (Table **1**) in more detail, striking differences can be seen in the susceptibility of bacteria or fungi from the same species to a particular NP. For example, discrepancies in strain sensitivities to the NPs, NPY and GMAP, as well as hemokinin-1, have been found against a panel of various clinical candidal [12] and *Pseudomonas aeruginosa* strains [34]. These observations are consistent with data from studies on human cationic peptides, such as the β-defensins (hBD2, hBD3), which demonstrate strain-selective activity against both aerobic and anaerobic bacteria and against yeast [35]. Discrepancies in strain sensitivities may arise when laboratory strains are compared with clinical isolates. In biochemical terms, these differences are likely to include modifications that reduce the negative charge of the cell surface. In the case of Gram-negative bacteria, such modifications usually include subtle differences in LPS composition, for example the acylation of the lipid A components of LPS or the addition of 4-aminoarabinose to the phosphate group on the lipid A backbone. In Gram-positive bacteria, which lack LPS, specific modifications of teichoic acids through the increased incorporation of significant quantities of D-alanine could give rise to increased positive surface charge [36]. Differences in strain susceptibility in fungi usually involve variation in the quantity of mannans in the outermost layer of the cell wall. For instance, the specific loss of negatively charged N-linked phosphomannans from cell wall proteins of *C. albicans* has been shown to induce resistance to cationic antimicrobial peptides [37].

Drawing analogies from research on conventional human antimicrobial peptides, additional mechanisms for strain susceptibility differences could involve external trapping of NPs or active efflux of NPs. Strain sensitivities could also arise as a result of variations in ATP leakage through a partially perforated cell membrane which could potentiate the killing effect [12]. Overall, the variability in NP susceptibilities observed suggests that a variety of strains should be tested in order to obtain a reliable profile of species susceptibility. 

#### 2.2.2 The Influence of Methods and Protocols 

The use of different experimental methods could lead to marked differences in the reported activity of NPs as even subtle changes in protocols can influence bioactivity. The most commonly employed antimicrobial assays to determine *in vitro *antimicrobial activity are radial diffusion assay or microbroth dilution assays. Radial diffusion assays [38] use a double-layer agarose diffusion method in which peptide is added to a well that is punched in a nutrient-poor agarose containing micro-organisms. After diffusion of the peptide, a nutrient rich overlay agarose is added and after overnight incubation, zones of bacterial growth inhibition are measured and converted to a minimum inhibitory concentration (MIC). In the liquid broth assay, suspensions of microorganisms in microtitre plate wells are treated with peptide and the MIC value is obtained by recording the concentration of peptide in those wells containing no visible growth. The minimum bactericidal concentration (MBC) can be calculated in the same experiment by determining the number of viable organisms in serially diluted aliquots of the treated bacterial suspensions. Alternatively the *lethal dose* that causes killing of at least 50% (LD_50_) of the target cells can be recorded. Less frequently, microbial growth may be measured using a spectrophotometer or luminometer to assess for example changes in cellular ATP production. The viability of parasites following NP treatment has been determined using viability-assessing dyes, for example the MTT reduction assay or trypan blue exclusion assay. 

One of the major factors that should be controlled carefully, irrespective of the method used for testing NP bioactivity is the ionic strength of the buffer. Divalent cations (e.g., Ca^2+^ and Mg^2+^) are known to contribute to bacterial outer membrane stabilization through their competition with cationic peptides for specific binding site on negatively charged moieties such as LPS. The physiologic concentrations of 150 mM NaCl and 1-2 mM Ca^2+^ and Mg^2+^ should therefore mimic the conditions of most human body fluids including serum, sputum and airway secretions. It is generally accepted however that these salt concentrations attenuate the activity of many cationic peptides [39]. Fortunately, as discussed in section 3.1.2, combinations of peptides may act synergistically to improve bioactivity under physiological salt concentrations [40]. Furthermore it is of interest to note that salt concentrations in saliva are much lower than 150 mM and thus it is appropriate to study peptide action in a range of buffer compositions.

The inoculum size and growth phase of the micro-organisms are also important in testing susceptibility to NPs. Since the activity of the NP is inversely related to the microbial load then the protocol should state clearly the OD or cell number (generally10^4^–10^7^ cells/ml). Furthermore the microbial growth phase is important as actively, exponentially growing cultures may have different susceptibilities to NPs compared with stationary, overnight cultures. Finally, the incubation time for the assay should be recorded as certain NPs may display only transient antimicrobial effects. In summary, factors such as ionic strength, pH, and viscosity (diffusion versus liquid assays) may affect antimicrobial activity assays and should be reported in full. Thus the lack of standard guidelines for testing NPs and other cationic peptides is a disadvantage for studying the direct antimicrobial activity of these compounds [41]. 

## 3 THE INDIRECT ANTIMICROBIAL ACTIVITY OF NEUROPEPTIDES 

NPs are released from nerve endings in response to various activating stimuli. As discussed above they can interact and eradicate pathogens by direct antimicrobial action. NPs are known to have a relatively short half -life *in vivo* since their replacement after release from nerve endings occurs via *de novo* synthesis. Therefore the formation of new peptides will result in a considerable delay before their levels in the nerve ending are restored [42]. This suggests that a sustained concentration of NPs *in vivo* may be difficult to achieve and as a result peptide levels may not reach sufficiently high concentration to decrease microbial load and overcome infection. However, NPs are likely to have much more sophisticated roles than simple antimicrobial action. It is possible therefore that the more important anti-infective roles of these compounds rely on their indirect immunomodulatory actions. 

### 3.1 Interaction with the Innate Immune System

The involvement of NPs as mediators of innate immune function has allowed the host to evolve intricate and overlapping mechanisms to control microbial invasion. To date the most intensely studied innate immune processes modulated by NPs are phagocytosis and the anti-infective inflammatory response. Both responses facilitate pathogen elimination and counteract infection by a complex series of self-limiting reactions designed to minimize the induction of excessive/chronic inflammation. Host responses are therefore tightly regulated to ensure a balance between protection and injury. NPs are recognized as extremely pleiotrophic immunomodulating macromolecules. The features of NP actions that are important in contributing to their complex immunomodulatory roles can be summarized by their ability to: (1) exert various, often contradictory effects, such as stimulation or inhibition of a given mechanism, and to show dual or opposing effects on particular cellular events, depending on dosage; (2) act in a concert with other inflammatory mediators or innate antimicrobials; (3) fulfill their function by means of specific receptors and also, paradoxically, utilize alternative receptors or non-receptor mediated mechanisms.

#### 3.1.1 Contradictory and Dual Effects of NPs in Phagocytosis

The complex and somewhat contradictory immunomodulatory effects of NPs are exemplified by their stimulation or inhibition of critical phagocytic events. The steps of phagocytosis such as chemotaxis, engulfment and reactive oxygen species production are differentially regulated by NP interaction with specific receptors. A good example is the distinct modulation of crucial functions of the neutrophil by various subtypes of the NPY receptor family. Y5 receptors have been shown to potentiate the production of reactive oxygen species whereas Y1 and Y2 receptors modify phagocytic engulfment [43]. Thus the definitive effect of a NP on a target cell will depend on which NP receptors are expressed by the cell and thus which NP-receptor signalling pathway is activated. It is worth adding that a similar dichotomy of NPY-receptor effects may also be observed within a single receptor subtype. For example, the Y1 receptor has been shown to be a strong negative regulator of T cells as well as key activator of APC function [44], suggesting a bimodal role for this receptor in the adaptive immune system. 

Like NPY, CGRP can also exert divergent effects on phagocytic function that depend on either direct (without opsonins) or indirect (with opsonins) pathogen recognition. Both NPY and CGRP exhibit a stimulatory effect on opsonin-dependent ingestion of pathogens by human granulocytes. Such an effect is not observed during opsonin-independent ingestion [21]. During phagocytosis, many parallel signaling pathways are activated simultaneously by a variety of receptors, which often function cooperatively [45]. Thus the effects of NPY and CGRP on opsonin-dependent phagocytosis appear to be a consequence of different receptor interplay in response to various modes of pathogen recognition. In contrast, an inhibitory effect for NPY has been observed in the phagocytosis of serum-opsonized targets by microglial cells [46]. Differences in the phagocytosis of opsonized targets by various cell types suggests that the Y1-dependent modulation of this cellular event may result in opposing effects depending on which effector cells are engaged in the process. The apparently conflicting regulation of phagocytosis by NPs may however reflect the most relevant defense strategy for the host. For example, since infection of macrophages is a pivotal step for replication of *Leishmania major*, then SP- or NPY-mediated inhibition of either the chemotaxis of phagocytes or the pathogen engulfment would reflect a protective action of the NP against this obligate intracellular parasite [47-49]. Conversely, the engulfment and phagocytosis of yeast by macrophages, which is potentiated in the presence of NPY or CGRP [50, 51], can be proposed to enhance antifungal defense. Although not part of the phagocytosis process *per se*, the inhibitory effects of VIP on leukocyte migration at high peptide concentration and its stimulatory effects at lower concentration [52] are additional examples of the functional duality of NPs in leukocyte action. 

#### 3.1.2 Neuropeptides and their Synergistic or Additive Effects in Inflammation

NPs are known to be potent modulators of the inflammatory response. Indeed the contribution of the nervous system to inflammation has been defined as the ‘neurogenic’ inflammatory response. SP, CGRP and NPY induce secretion of key proinflammatory cytokines, chemokines and arachidonic acid metabolites from immune and non-immune cells whereas VIP, α-MSH and AM tend to exert the opposite effects [53-64] (Fig. (**2**)). The communication between the immune and nervous systems is bi-directional, therefore cytokines may influence the nervous system and NPs may influence immune cell function. The potential for cytokines to regulate the neurogenic inflammatory response has been studied in a variety of tissues, including oral tissues, which are extensively innervated by peptidergic nerves. In dental pulp tissue, upregulation of NPY-specific Y1 receptor expression has been shown in the presence of inflammatory cytokines [65] and in dental caries [66]. Dental pulp fibroblasts have been shown to express SP and the NK-1 receptor [67]. The opposing effects reported for IL-1β and TGF-β1 on SP and NK-1 receptor expression reflect the antagonistic roles of these two cytokines in pulpal inflammation and support cross-talk between immune and nervous system components. 

In order to intensify their indirect action, NPs may act in concert with other inflammatory mediators or innate immune molecules. LPS has been shown to enhance SP-mediated neutrophil adherence to lung epithelial cells and release of proinflammatory cytokines such as IL-1β and TNF-α [68]. NPY and SP can indirectly activate epithelial cells to produce classical antimicrobial peptides such as cathelicidins or β-defensins [69]. These certain host peptides exert bactericidal and fungicidal activities against a broad range of pathogens [35, 70]. There is also clear indication that SP and CGRP exert a synergistic effect in augmenting the production of TNF-α from human peripheral blood mononuclear cells [53], thereby orchestrating the inflammatory reaction. Interaction between AM and factor H, an important complement regulator, strengthens evidence for the proposed cooperation between NPs and key components of the innate immune system. The resultant AM-factor H complex has been shown to modify the bioactivity of both molecules. Factor H seems to protect AM from enzymatic degradation by matrix metalloproteinase 2, thus increasing the peptide’s bioavailability. This protection against enzymatic action is thought to be afforded as a result of steric hindrance that masks potential susceptible enzyme cleavage sites. However this effect also appears to diminish the antimicrobial activity of AM presumably by steric interference with bacterial membrane interaction. The increased bioavailability and decreased antimicrobial activity in the presence of its binding partner further support the complexity of action of AM in inflammation. The formation of AM-factor H complex also appears to modulate the actions of factor H. When AM binds to factor H then the ability of factor H to facilitate the cleavage of complement component C3b in the presence of factor I is increased, thereby enhancing opsonisation [71, 72]. More recently combinations of VIP or the hypothalamic NP orexin (ORXB) with the human cathelicidin LL-37 facilitated bactericidal effects against *Escherichia coli*, *Pseudomonas aeruginosa*, *Streptococcus mutans* and *Staphylococcus aureus *under physiological salt concentrations [40]. These findings indicate that peptides may work together to facilitate pathogen elimination *in vivo* and emphasize a multifunctional role for NPs as modulators of innate immune defense.

#### 3.1.3 Receptor Dependent and Independent Mechanisms

NPs generally perform their regulatory cellular effects by activating specific G-protein coupled receptors and distinct patterns of receptor expression typically result in their temporal activation in infectious sites, (Table **2**). For example, in the activation of mast cells, confirmed engagement of specific receptors for SP and VIP has been described in the processes of mast cell degranulation and chemokine production [57]. However, the induction of mast cell degranulation by SP can also involve receptor-independent activation. It is thought that mast cell degranulation is initiated by binding of SP to negatively charged sites on the plasma membrane, followed by intracellular translocation of peptide in an energy-independent fashion. Once inside the cell SP directly stimulates G proteins and induces granule exocytosis [56]. The receptor-independent mechanism of mast cell degranulation and histamine release has also been proposed for AM [73]. In addition to receptor-independent mechanisms, some NPs utilize “non specific” receptor mechanisms. An excellent example is the activation of macrophages by CGRP through ligation of mannose receptors [51], which confirms a role for CGRP in lectin-mediated phagocytosis. The expression of functional CGRP-specific receptors on mature human neutrophils (PMNs) has yet to be confirmed [74], however CGRP can directly activate these cells leading to lactoferrin release from secondary granules [75]. Secondly, the activation of engulfment and ROS generation, both critical steps in PMN phagocytosis, are considerably intensified in the presence of CGRP [21]. These results support the involvement of receptor-independent mechanisms in NP-induced activation of professional phagocytes. A common theme emerging from the examples discussed above is that at least some NPs are involved in non-classical routes of cell activation that are independent of specific receptors. 

### 3.2 Interaction with the Adaptive Immune System

The modulation of adaptive immunity by NPs, involves both cell-mediated and antibody-mediated immune responses, (Fig. (**2**)). 

#### 3.2.1 Modulation of Cell-Mediated Immunity

NPs modulate Th1/Th2 cytokine secretion [76], mobilization of immature dendritic cells [77] and the cytotoxic capacity of CD8^+^ T lymphocytes [78]. Thus, NPs can shift both the defense response directed by the relevant subpopulations of T helper cells and enhance cellular mechanisms responsible for priming the T cell response, antigen recognition and effector phase of adaptive cellular immunity. The anti-infective properties of NPs may have important implications in diseases such as asthma or chronic obstructive pulmonary disease in which a Th1/Th2 imbalance is observed and where microbial participation has been postulated [79, 80]. Additionally, CGRP, NPY, and SOM can regulate integrin-mediated T cell adhesion to the extracellular matrix (ECM) ligand fibronectin via activation of β1 integrins on the surface of lymphocytes [81]. This acquired ability allows T cells to migrate, extravasate and therefore gain access to the site of injury, infection or inflammation. Since T cell influx to the site of infection generally takes place after primary neutrophil recruitment [82], the aforementioned activities of NPs on T cells could be particularly important in eradication of intracellular pathogens such as Mycobacteria or viruses. 

#### 3.2.2 Modulation of Antibody-Mediated Immunity

Some NPs have also been shown to modulate humoral adaptive immune response. For example SP, neurokinin A (NKA) and neurokinin B (NKB) can modulate immunoglobulin isotype production by augmenting IgG_3_, IgG_4_ and IgA_2_ synthesis. SP, NKA and NKB can also induce a shift from IgA to IgG production in the mucosa [83]. Since the structure of immunoglobulins is strictly connected with their functionality, then the regulatory effect of NPs on immunoglobulin class/subclass synthesis may lead to the activation of different antibody-mediated effector mechanisms. Interestingly, SP and VIP differentially stimulate production of IgA and IgM by cells from the spleen, Peyer’s patches and mesentheric lymph nodes [84]. Given that the innervation and thus the availability of sensory and autonomic NPs may modulate the quality and/or quantity of immunoglobulin production then the site of pathogen entry should be considered important in determining the role of NPs in generating the most relevant and protective immune response. 

## 4 THERAPEUTIC POTENTIAL IN THE LIGHT OF *IN VIVO* STUDIES

The rising incidence of bacterial resistance is a driving force for the development of new anti-infective strategies. Some of these strategies include the use of antimicrobial peptides, or their mimetics in animal models to determine their potential antimicrobial and/or immunomodulatory activity *in vivo* [85, 86]. Future approaches for the use of anti-infective therapeutics could involve novel peptides: (i) as single anti-infective agents with direct antimicrobial action; (ii) in combination with conventional antibiotics or antivirals to promote additive or synergistic effects; (iii) as immunostimulatory agents that enhance natural innate or adaptive immunity and (iv) as endotoxin-neutralizing agents [87]. 

Therapeutic applications of NPs have been studied in the treatment of some severe inflammatory and autoimmune disorders and detailed overviews on this topic are available elsewhere [88, 89]. However, little information is available regarding the potential for NP antimicrobial/anti-infective therapy in humans. By drawing analogies with synthetic analogs of classical host defense peptides such as magainin 2 (pexiganan, MSI-78), indolicidin (omiganan, MX-226/MBI-226), protegrin 1 (iseganan, IB-367) or histatin (PAC-113), which have been extensively examined and used as anti-infective therapies in human clinical trials (Phase II or III) [86], it seems that the potential usefulness of NPs as direct antimicrobial agents may be limited. Many of the analogs of classical host defense peptides tested in clinical trials have rather weak direct antimicrobial potency, which does not correlate with the strong effectiveness observed in *in vitro* studies. Therefore they appear to demonstrate few advantages over existing antibiotic-based therapies and have not received FDA approval for clinical use. However, one human bactericidal/permeability-increasing protein rBPI_23_ (Neuprex) has been introduced for the treatment of meningococcal sepsis [90]. Regarding peptides derived from the neuroendocrine system, even fewer clinical trials have tested their antimicrobial effectiveness. To date, only two peptides, namely an α-MSH derivative and ghrelin have entered Phase II clinical trials for the treatment of vulvovaginal candidiasis and chronic respiratory infection, respectively. Encouragingly, positive anti-candidal efficacy has been reported for the α-MSH derivative and anti-inflammatory efficacy has been reported for both compounds [86].

### 4.1 NPs in Bacterial Infections

Despite the lower direct killing potency of NPs compared with conventional antimicrobial peptides such as defensins and cathelicidins, it is still advisable to explore NPs for their immunomodulatory properties. The fundamental role of host defense peptides in maintaining host homeostasis can shed light on potential roles for NPs in immunomodulation. A connection between deficiency of host defense peptides and increased microbial colonization has been deduced from pathologies such as Crohn’s disease which is associated with reduced expression of α-defensins in the small intestine [91] and atopic dermatitis which is linked with reduced expression of LL-37, hBD2 and hBD3 [92]. A possible protective role for NPY was suggested in patients with periodontal inflammation who have significantly lowered levels of this peptide in diseased gingival crevicular fluid compared with healthy sites. The functionality of NPY was supported in this study by the presence of Y1 receptors in local gingival tissue [93]. Significantly decreased levels of NPY have also been observed in patients suffering from inflammatory bowel disease (IBD) [94]. In the case of AM, increased levels of AM have been reported in gingival crevicular fluid from periodontitis patients, which may reflect increased expression from epithelial cells as a result of stimulation by LPS from periodontal pathogens [3, 95, 96]. In fact the levels of AM present in gingival crevicular fluid predict its direct antibacterial action [8], supporting a physiologically relevant role for AM in the gingival crevice. 

Interestingly, altered NP gene expression may be connected in part with predisposition to bacterial infection. In pediatric bronchitis, bacterial colonization of the lower airway with *Streptococcus pneumonia*, *Haemophilus influenza* and *Moraxella catarrhalis* was shown to be associated with reduced expression of TAC1, the preprotachykinin gene encoding SP [97]. Further evidence confirming the *in vivo* efficacy of NPs has been reported in animal models in which SP was shown to regulate IFN-γ production by natural killer cells following NK1 receptor engagement. This modulatory activity of SP was linked to the resolution of corneal infection caused by *Pseudomonas aeruginosa *[98]. The upregulation of AM expression in response to *Escherichia coli *o*r Mycobacterium paratuberculosis* infection in animal models was suggested to enhance mucosal immunity [99]. Exogenously applied AM, in turn, has been shown both *in vitro* and *in vivo* to protect the ileum by reducing staphylococcal α-toxin-induced microcirculatory disturbances as well as reducing intestinal epithelial permeability [100, 101]. This AM-mediated improvement of gut mucosal barrier function appears to prevent the detrimental translocation of bacteria and their products from the gut lumen into surrounding tissues [102]. In the case of α-MSH, the indirect antibacterial effects are connected with multiple general anti-inflammatory properties. For example, in human keratinocytes α-MSH down-regulates β-1 integrins and heat shock surface protein 70, both of which are essential molecules in the invasion of keratinocytes by *S. aureus *[103]. Furthermore α-MSH down-regulates pro-inflammatory cytokine expression in human keratinocytes suggesting it has a protective role in both infection and inflammation [103]. It is therefore tempting to speculate that taken together these data suggest a relationship between resolution of infection *in vivo* and the presence of action of NPs.

### 4.2 NPs in Sepsis

The principal injurious consequence of the host response to bacteria is inflammation, which can lead to the clinical symptoms associated with septic shock such as fever, systemic inflammatory response, organ dysfunction and hypertension if the pathogen is not eradicated. One of the most powerful mediators of tissue damage in endotoxic shock is TNF-α [104]. A great body of evidence supports a protective role for selected NPs in bacterial sepsis, which is conferred in part by TNF-α inhibition and endotoxin neutralization. It has been shown that α-MSH modulates serum TNF-α as well as inducible nitric oxide synthase activity in the lungs and liver of mice with endotoxemia after central injection of NP [105]. In a model of polymicrobial sepsis, mice treated with the α-MSH analogue AP_214_ showed ameliorated severe septic shock and sepsis-induced acute kidney injury and mortality [106]. A reduction in TNF-α and IL-6 concentrations, along with significantly increased survival, was observed following VIP application in an intraperitoneal injection animal model of endotoxic shock [107], supporting VIP’s broad anti-inflammatory action [108]. In a cecal ligation and puncture animal model of sepsis, VIP was shown to retain it protective effects against septic lethality even if administered 24 hours after sepsis induction [109]. Very recently however, VIP knock-out mice have been shown to exhibit resistance to LPS-induced entodoxemia. Levels of TNFα and IL-6 mRNA were decreased in serum and peritoneal suspensions in LPS-treated VIP knock-out mice, suggesting that the ability of myeloid cells to elicit an inflammatory response when exposed to LPS may be impaired in the chronic absence of VIP [110]. The complexity of VIP action in sepsis is yet another example of NP duality. Therefore the discussed here *in vivo* effects of VIP deserve further investigation with regard to its time and mode of administration as well as mechanism of action. The actions of ghrelin in a mouse model of sepsis resulted in improved bacterial clearance *in vivo*, accompanied by bactericidal effects *in vitro*. The deactivation of resident and infiltrating macrophages was proposed to be the key mechanism in the therapeutic effect of the neuropeptide [111]. The therapeutic usefulness of AM has been confirmed using animal models in which exogenously applied AM reduced mortality in endotoxin- [112] and exotoxin-related [113] shock responses. In addition, NPY administered peripherally was responsible for long-lasting stabilization of body temperature and prevented hypotension in endotoxemic rodents [114]. Although evidence from animal models indicates that systemically amplified inflammatory cascades may be silenced in part by NPs, their potential efficacy in sepsis remains to be determined in clinical trials.

### 4.3 NPs in Viral Infections

As broad spectrum antimicrobials, NPs may also have a role in defense against viral infections. A protective role for NPY in retrovirus-induced neurological disease has been reported and is thought to involve regulation of the entry of virus-infected cells into the central nervous system (CNS) [115]. SP has been shown to block neuronal spread of measles virus that can also infect the CNS. This role was confirmed by reduced infection of susceptible mice following both genetic deletion and pharmacological inhibition of the NK-1 receptor [116]. SP and CGRP, in turn, have been shown to enhance the macrophage-mediated inflammatory response to HSV-1 through secretion of the pro-inflammatory cytokines IL-1β and TNF-α [117]. Furthermore, therapeutic treatment with CGRP abolished airway hyperresponsiveness following respiratory syncytial virus (RSV) infection, suggesting a protective role for CGRP in the development of RSV-induced airway dysfunction [118]. The increased cytolytic potential of CD8^+^ T cells in response to stimulation with α-MSH has been recently demonstrated. It was evidenced by upregulation of genes encoding for cytolytic enzymes such as granzyme A, granzyme B and perforin as well as those engaged in apoptosis induction such as Fas [78]. Since cytotoxic CD8^+^ T cells are critically involved in antiviral defense, this finding may suggest a possible role for α-MSH in antiviral therapy. 

The protective effects of NPs in one disorder may be manifest as deleterious effects in another. It has been demonstrated that SP enhances HIV infection of peripheral blood monocyte-derived macrophages [119] and activates HIV-1 replication in latently infected cells [120]. This destructive role for SP in the pathogenesis of HIV was confirmed also by the antiretroviral action of the NK-1 receptor antagonist (aprepitant) that inhibited drug-resistant HIV infection of macrophages *in vitro* [121]. Furthermore, in HIV infection, VIP has been shown to activate transcription of the HIV LTR promoter, whereas the VIP receptor VPAC-1 plays a significant role in facilitating the successful viral cDNA integration into host genome [122]. 

Several independent lines of evidence from *in vivo* studies support further study of the multifunctionality of NP action in microbial-associated diseases. The divergent actions of NPs in microbial diseases should be taken into account when considering the theoretical advantages and disadvantages of NPs as antimicrobial agents, (Table **3**).

## CONCLUSIONS

Evidence to date would suggest that the direct antimicrobial actions of the majority of NPs appear not to be as effective as their antibiotic counterparts indicating that this aspect of their therapeutic usefulness remains elusive. A notable exception is however α-MSH which has proved to be successful in clinical trials. Future research on peptide mimetics could overcome the initial difficulties encountered with many of the other NPs studied. Considering their immunomodulatory role, NPs have been shown to modulate a wide range of host defense mechanisms such as attraction of phagocytic cells to the site of infection, modulation of critical phagocytic function, triggering pro-inflammatory mediators and regulation of T or B cell function following antigenic stimulation. Despite their complexity of action, there are several key advantageous to the development of NP-based drug analogs or receptor antagonist: (i) “milder” blocking effects as a result of their ability to modulate rather than control the immune response; (ii) limited side effects (since they are natural molecules with a finite half life); (iii) pleiotropic functions which allow multiple targeted effects and (iv) receptor expression and distribution should limit off-target agonist or antagonist action [42]. 

In conclusion the pleiotropic nature of NPs indicates that they may yield pro-inflammatory and anti-inflammatory effects as well as pro-infective and anti-infective effects. Although a pro-infective response in never desirable, it is tempting to suggest that a short-lived pro-inflammatory response will increase efficiency in eliminating infective agents during the early stages of inflammation. Pro-inflammatory effects must however be controlled so that they do not contribute to the development or exacerbation of chronic inflammatory disorders such as periodontitis, asthma and chronic obstructive pulmonary disease. In some diseases a neurogenic contribution to inflammation has already been proposed [123] and thus it is imperative that future research should be directed towards elucidating and enhancing beneficial inflammatory responses driven by NPs without potentiating chronic inflammation. In summary, although NPs are recognized as important components of antimicrobial innate immunity, their value as the therapeutic agents remains to be fully elucidated. Thus we are tasked with greater research effort in this field to fully realize the anti-infective, antimicrobial and immunomodulatory properties of NPs as additional weapons in the fight against infection. 

## Figures and Tables

**Fig. (2):**
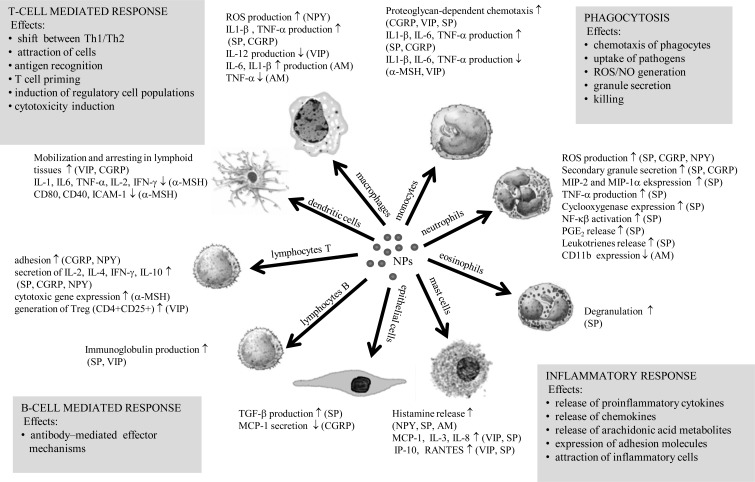
Overview of the effects exerted by neuropeptides on key cells engaged in host defense and their possible implications for innate and adaptive immune responses. Data derived from references [50, 53-64, 72, 76-78, 141, 172-179].

**Table 1.** The Direct Antimicrobial Activities of Selected Human Neuropeptides Against Various Strains of Bacteria, Fungi and Protozoa Parasites Within the Species Listed

NEUROPEPTIDES	SP	NPY (NPY_13-36_)	CGRP	VIP	AM	α-MSH	References
**Bacteria, Gram negative**							
*Escherichia coli*	4.2 - 400 µM	4.2 - 11 µM	0.55 µM	1.5 µM	0.06 µM	1 - 100 µM*	[8-11, 124]
*Klebsiella pneumoniae*	>370 µM	>117 µM	-	-	-	-	[ 9]
*Proteus vulgaris*	>370 µM	-	-	-	-	-	[9]
*Pseudomonas aeruginosa*	11.7 - >370 µM	31 - >117 µM	1.5 µM	1.2 µM	-	-	[9-11]
*Moraxella catarrhalis*	100 µM*	100 µM* (8 - 12.5 µM)	100µM*	-	-	-	[21]
*Haemophilus influenzae*	>370 µM*	100 µM* (25 - 30 µM)	100µM*	-	2 µM	-	[8, 21]
*Aeromonas caviae*	-	14 µM	-	-	-	-	[13]
**Bacteria, Gram positive**							
*Staphylococcus aureus*	50 - 370 µM	>117 µM	>132 µM	>150 µM	2 µM	1 pM*- 100 µM*	[7-11, 18, 124]
*Enterococcus faecalis*	>370 µM	4.6 - 29 µM	>132 µM	>150 µM		-	[9, 11, 13]
*Streptococcus mutans*	128 - >370 µM	49 - >117 µM	>132 µM	45 µM	2 µM	-	[8, 10, 11]
*Actinobacillus actinomycetemcomitans*	>370 µM	>117 µM	-	-	-	-	[10]
*Lactobacillus acidophilus*	55 µM	66 µM	>117 µM	>150 µM	-	-	[11]
*Nocarida brasiliensis*	-	7 µM*	-	-	-	-	[13]
**Fungi**							
*Candida albicans*	6 - >370 µM	5.6 - > 57 µM (1 - 2 µM)	16.6 µM	13.9 µM		1 -100 µM*	[6, 7, 9, 11, 13, 125]
*Candida krusei*	-	(4 - 8 µM)*	-	-	-	-	[12]
*Candida tropicalis*	-	(4 - 8 µM)*	-	-	-	-	[12]
*Candida utilis*	-	(4 - 8 µM)*	-	-	-	-	[12]
*Cryptococcus neoformans*	-	5.7 - 7 µM	-	-	-	-	[13]
*Arthroderma simii*	-	3.4 - 4.7 µM	-	-	-	-	[13]
**Parasites**							
*Trypanosoma brucei*	-	-	-	3 µM*	1.8 µM*	9 µM*	[14, 29]
*Leishmania major*	-	5.8 µM*	-	-	-	-	[13]

* Refers to ≥ LD_50_ - (the dose that causes at least 50% killing). In other cases minimal inhibitory concentration (MIC) was measured.

“-” means no data available.

**Table 2.** Expression of Neuropeptide-Specific Receptors on Immune and Non-Immune Cells.

CELLS	NEUROPEPTIDES	RECEPTORS	References
**Neutrophils**	NPY	Y1	[43]
		Y2	[43]
		Y4	[43]
		Y5	[43]
	SP	NK1R	[126]
	VIP/PACAP	VPAC1	[127]
	α-MSH	MC-1	[128]
**Monocytes**	NPY	Y2	[129]
	SP	NK1R	[130]
	AM	CRLR-RAMP2	[131]
		CRLR-RAMP3	[131]
	VIP/PACAP	VPAC1	[132, 133]
		VPAC2	[133, 134]
		PAC1	[133]
	α-MSH	MC-1	[135, 136]
		MC-3	[137]
		MC-5	[137]
**Macrophages**	NPY	Y1	[138]
	SP	NK-1R	[130]
	AM	CRLR-RAMP2	[131]
		CRLR-RAMP3	[131]
	CGRP	CGRP-R	[139]
	VIP/PACAP	VPAC1	[140]
		VPAC2	[141, 142]
		PAC1	[143]
	α-MSH	MC-1	[144, 145]
**Lymphocytes B**	SP	NK1R	[146]
	α-MSH	MC-1	[145]
**Lymphocytes T**	SP	NK1R	[147]
	AM	CRLR-RAMP2	[131]
		CRLR-RAMP3	[131]
	CGRP	CGRP-R	[148]
	VIP/PACAP	VPAC1	[132]
		VPAC2	[132]
	α-MSH	MC-1	[145]
**Eosinophils**	VIP/PACAP	VPAC1	[149]
**Mast cells**	SP	NK1R	[57, 150]
		NK2R	[57]
		NK3R	[57]
	VIP/PACAP	VPAC1	[151]
		VPAC2	[57, 151]
	α-MSH	MC-1	[152]
**Dendritic cells**	SP	NK-1R	[153]
	AM	CRLR-RAMP2	[131]
		CRLR-RAMP3	[131]
	CGRP	CGRP-R	[154]
	VIP/PACAP	VPAC1	[131]
	α-MSH	MC-1	[155]
**Natural killers**	SP	NK1R	[156]
	α-MSH	MC-1	[145]
**Fibroblasts**	NPY	Y1	[65]
	SP	NK1R	[157]
	AM	L1-R	[158]
**Keratinocytes**	SP	NK1R	[157]
	AM	L1-R	[158]
	VIP/PACAP	VPAC1	[159, 160]
		VPAC2	[159, 160]

**Table 3.** Advantages and Disadvantages of Antimicrobial Therapy with NPs or NP Analogues

Benefits
rapid and easy access to sites of infection as results of small size and amphipathicity effective removal by host metabolism various mechanisms of direct antimicrobial action and possibility to target more than one specific pathway limited bacterial resistance generation connected with their mechanism of action modulation of key host antimicrobial defense mechanisms weak immunogenicity connected with their physiologic origin relatively facile synthesis with the option of modification
**Drawbacks**
low microbicidal activity that requires high therapeutic doses possibility of undesirable effects as a result of their complex actions high sensitivity to various tissue and microbial proteases

## LIST OF ABBREVIATIONS

AM: = Adrenomedullin
CGRP: = Calcitonin-gene related peptide
IL: = Interleukin
IP-10: = IFN-γ inducible protein
LPS: = Lipopolysaccharide
MCP-1: = Monocyte chemoattractant protein-1
α-MSH: = Melanocyte-stimulating hormone
NF-κβ: = Nuclear factor κβ
NP: = Neuropeptide
NKA: = Neurokinin A
NKB: = Neurokinin B
NPY: = Neuropeptide Y
PGE_2_: = Prostaglandin E_2_
RANTES: = Regulated on activation normal T cell expressed and secreted chemokine
ROS: = Reactive oxygen species
SP: = Substance P
TGF-β: = Transforming growth factor β
TNF-α: = Tumor necrosis factor α
VIP: = Vasoactive intestinal peptide.
